# 3β,12β,14α-Trihydroxy­pregnan-20-one

**DOI:** 10.1107/S1600536809013853

**Published:** 2009-04-25

**Authors:** Hefang Shi, Yingxia Li

**Affiliations:** a Key Laboratory of Marine Drugs, Ministry of Education of China, School of Medicine and Pharmacy, Ocean University of China, Qingdao 266003, People’s Republic of China

## Abstract

The title compound, C_21_H_34_O_4_, is a steriod of the pregnane family prepared by the sequential oxidation and reduction of 3β,12β-diacet­oxy-20-ethyl­enedioxy­pregnan-14-ene. The con­formations of the six-membered rings are close to chair forms, while the five-membered ring adopts an envelope conformation. All the rings are *trans*-fused and an intra­molecular O—H⋯O hydrogen bond occurs. In the crystal structure, inter­molecular O—H⋯O hydrogen bonds link the mol­ecules into a two-dimensional network.

## Related literature

For the synthesis, see: Templeton & Yan (1992 ▶); Fell & Heathcock (2002 ▶). For background on hecogenin, see: Ranu & Samanta (2003 ▶).
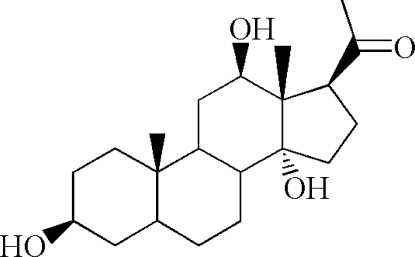


## Experimental

### 

#### Crystal data

C_21_H_34_O_4_
*M* *_r_* = 350.48
Monoclinic, 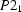

*a* = 6.1364 (7) Å
*b* = 12.1472 (13) Å
*c* = 12.7593 (14) Å
β = 101.513 (2)°
*V* = 931.94 (18) Å^3^
*Z* = 2
Mo *K*α radiation
μ = 0.08 mm^−1^
*T* = 293 K
0.50 × 0.44 × 0.32 mm

#### Data collection

Bruker SMART CCD area-detector diffractometer
Absorption correction: multi-scan (*SADABS*; Bruker, 1998 ▶) *T*_min_ = 0.729, *T*_max_ = 0.97
5569 measured reflections
2194 independent reflections
1930 reflections with *I* > 2σ(*I*)
*R*_int_ = 0.107

#### Refinement

*R*[*F* ^2^ > 2σ(*F* ^2^)] = 0.048
*wR*(*F* ^2^) = 0.125
*S* = 1.01
2194 reflections
238 parameters
1 restraint
H atoms treated by a mixture of independent and constrained refinement
Δρ_max_ = 0.28 e Å^−3^
Δρ_min_ = −0.24 e Å^−3^

### 

Data collection: *SMART* (Bruker, 1998 ▶); cell refinement: *SAINT* (Bruker, 1998 ▶); data reduction: *SAINT*; program(s) used to solve structure: *SHELXS97* (Sheldrick, 2008 ▶); program(s) used to refine structure: *SHELXL97* (Sheldrick, 2008 ▶); molecular graphics: *SHELXTL* (Sheldrick, 2008 ▶); software used to prepare material for publication: *SHELXTL*.

## Supplementary Material

Crystal structure: contains datablocks I, global. DOI: 10.1107/S1600536809013853/hb2940sup1.cif

Structure factors: contains datablocks I. DOI: 10.1107/S1600536809013853/hb2940Isup2.hkl

Additional supplementary materials:  crystallographic information; 3D view; checkCIF report

## Figures and Tables

**Table 1:** Hydrogen-bond geometry (Å, °)

*D*—H⋯*A*	*D*—H	H⋯*A*	*D*⋯*A*	*D*—H⋯*A*
O3—H3⋯O4^i^	0.81 (4)	2.19 (4)	2.916 (3)	150 (3)
O2—H2⋯O4	0.80 (4)	2.01 (4)	2.771 (3)	158 (3)
O1—H1⋯O2^ii^	0.88 (5)	2.05 (6)	2.928 (3)	170 (4)

Symmetry codes: (i) 
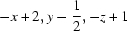
; (ii) 
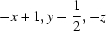
.

**Fig. 1.**
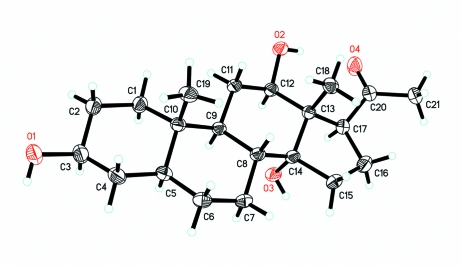
The molecular structure of (I) with displacement ellipsoids for the non-H atoms drawn at the 50% probability level.

**Fig. 2.**
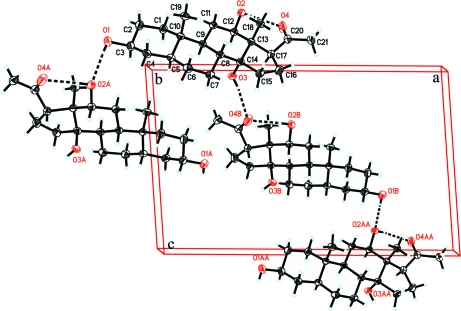
The molecular packing of (I) viewed down the a axis. Dashed lines indicate hydrogen bonds.

## supplementary crystallographic information

### Comment

The title compound was obtained by oxidation and reduction of the corresponding 3β,12β-diacetoxypregnan-20-ethylenedioxy-14-ene, which was prepared from hecogenin (Fell & Heathcock, 2002; Ranu & Samanta, 2003). We have undertaken the X-ray crystal structure determination of (I) in order to establish its molecular conformation and relative stereochemistry.

The hydroxyl group at C12 and the acetyl group at C17 are beta-oriented respectively, whereas the hydroxyl group at C14 is alpha-oriented (Fig. 1). The conformation of the six-membered rings in both molecules are close to chair forms, while the five-membered ring adopts an envelope conformation. All rings in both molecules are *trans*-fused. The molecules of (I) are held together by an extensive O—H···O hydrogen-bonding two-dimensional network (Table 1, Fig. 2).

### Experimental

The title compound was prepared according to the literature method (Fell & Heathcock, 2002; Templeton & Yan 1992). Crystals suitable for X-ray analysis were obtained by slow evaporation of a methanol solution at room temperature (m.p. 343–347 K).

### Refinement

Anomalous dispersion was negligible and Friedel pairs were merged before refinement.  The O-bond H atoms were located in a difference map and their positions were freely refined with *U*_iso_(H) = 1.5U_eq_(O). The C-bound H atoms were fixed geometrically at ideal positions (C—H = 0.96–0.98 Å) and refined as riding with *U*_iso_(H) = 1.2U_eq_(C) or 1.5U_eq_(methyl C).

### Crystal data

**Table:**

C_21_H_34_O_4_	*F*(000) = 384
*M**_r_* = 350.48	*D*_x_ = 1.249 Mg m^−^^3^
Monoclinic, *P*2_1_	Melting point: 431(2) K
Hall symbol: P 2yb	Mo *K*α radiation, λ = 0.71073 Å
*a* = 6.1364 (7) Å	Cell parameters from 2719 reflections
*b* = 12.1472 (13) Å	θ = 4.7–55.9°
*c* = 12.7593 (14) Å	µ = 0.08 mm^−^^1^
β = 101.513 (2)°	*T* = 293 K
*V* = 931.94 (18) Å^3^	Prism, colourless
*Z* = 2	0.50 × 0.44 × 0.32 mm

### Data collection

**Table:**

Bruker SMART CCD area-detector diffractometer	2194 independent reflections
Radiation source: fine-focus sealed tube	1930 reflections with *I* > 2σ(*I*)
graphite	*R*_int_ = 0.107
ω scans	θ_max_ = 27.5°, θ_min_ = 2.3°
Absorption correction: multi-scan (*SADABS*; Bruker, 1998)	*h* = −7→7
*T*_min_ = 0.729, *T*_max_ = 0.97	*k* = −15→13
5569 measured reflections	*l* = −16→13

### Refinement

**Table:**

Refinement on *F*^2^	Primary atom site location: structure-invariant direct methods
Least-squares matrix: full	Secondary atom site location: difference Fourier map
*R*[*F*^2^ > 2σ(*F*^2^)] = 0.048	Hydrogen site location: inferred from neighbouring sites
*wR*(*F*^2^) = 0.125	H atoms treated by a mixture of independent and constrained refinement
*S* = 1.01	*w* = 1/[σ^2^(*F*_o_^2^) + (0.0733*P*)^2^] where *P* = (*F*_o_^2^ + 2*F*_c_^2^)/3
2194 reflections	(Δ/σ)_max_ < 0.001
238 parameters	Δρ_max_ = 0.28 e Å^−^^3^
1 restraint	Δρ_min_ = −0.24 e Å^−^^3^

### Special details

**Table:**

Geometry. All e.s.d.'s (except the e.s.d. in the dihedral angle between two l.s. planes) are estimated using the full covariance matrix. The cell e.s.d.'s are taken into account individually in the estimation of e.s.d.'s in distances, angles and torsion angles; correlations between e.s.d.'s in cell parameters are only used when they are defined by crystal symmetry. An approximate (isotropic) treatment of cell e.s.d.'s is used for estimating e.s.d.'s involving l.s. planes.
Refinement. Refinement of *F*^2^ against ALL reflections. The weighted *R*-factor *wR* and goodness of fit *S* are based on *F*^2^, conventional *R*-factors *R* are based on *F*, with *F* set to zero for negative *F*^2^. The threshold expression of *F*^2^ > σ(*F*^2^) is used only for calculating *R*-factors(gt) *etc*. and is not relevant to the choice of reflections for refinement. *R*-factors based on *F*^2^ are statistically about twice as large as those based on *F*, and *R*- factors based on ALL data will be even larger.

### Fractional atomic coordinates and isotropic or equivalent isotropic displacement parameters (Å^2^)

**Table:**

	*x*	*y*	*z*	*U*_iso_*/*U*_eq_	
O1	0.2262 (4)	−0.0588 (2)	−0.28839 (15)	0.0605 (6)	
H1	0.152 (7)	−0.121 (5)	−0.292 (3)	0.091*	
O2	0.9924 (3)	0.22625 (16)	0.32210 (14)	0.0440 (4)	
H2	1.062 (6)	0.223 (4)	0.382 (3)	0.066*	
O3	0.7898 (3)	−0.09548 (15)	0.33306 (15)	0.0455 (5)	
H3	0.767 (6)	−0.146 (4)	0.371 (3)	0.068*	
O4	1.1201 (4)	0.2028 (2)	0.54187 (16)	0.0695 (7)	
C1	0.5918 (4)	0.0590 (2)	−0.04305 (19)	0.0439 (6)	
H1A	0.6605	0.1311	−0.0317	0.053*	
H1B	0.7099	0.0046	−0.0316	0.053*	
C2	0.4657 (5)	0.0512 (3)	−0.1588 (2)	0.0503 (6)	
H2A	0.3593	0.1111	−0.1735	0.060*	
H2B	0.5695	0.0587	−0.2066	0.060*	
C3	0.3446 (5)	−0.0573 (2)	−0.18001 (19)	0.0476 (6)	
H3A	0.4549	−0.1166	−0.1704	0.057*	
C4	0.1926 (4)	−0.0747 (2)	−0.1019 (2)	0.0457 (6)	
H4A	0.1198	−0.1456	−0.1150	0.055*	
H4B	0.0785	−0.0182	−0.1124	0.055*	
C5	0.3237 (4)	−0.07015 (19)	0.01358 (19)	0.0384 (5)	
H5	0.4398	−0.1265	0.0194	0.046*	
C6	0.1835 (5)	−0.1005 (2)	0.0945 (2)	0.0479 (6)	
H6A	0.1104	−0.1704	0.0748	0.057*	
H6B	0.0693	−0.0451	0.0937	0.057*	
C7	0.3245 (5)	−0.1090 (2)	0.2067 (2)	0.0470 (6)	
H7A	0.4239	−0.1715	0.2100	0.056*	
H7B	0.2283	−0.1216	0.2573	0.056*	
C8	0.4612 (4)	−0.0048 (2)	0.23833 (18)	0.0355 (5)	
H8	0.3577	0.0551	0.2449	0.043*	
C9	0.5946 (4)	0.02912 (18)	0.15366 (17)	0.0317 (5)	
H9	0.6985	−0.0312	0.1492	0.038*	
C10	0.4450 (4)	0.04102 (19)	0.04009 (18)	0.0345 (5)	
C11	0.7364 (4)	0.1310 (2)	0.19024 (19)	0.0359 (5)	
H11A	0.8319	0.1447	0.1396	0.043*	
H11B	0.6385	0.1939	0.1883	0.043*	
C12	0.8806 (4)	0.12266 (18)	0.30152 (19)	0.0348 (5)	
H12	0.9922	0.0651	0.3010	0.042*	
C13	0.7415 (4)	0.09215 (19)	0.38462 (19)	0.0348 (5)	
C14	0.6210 (4)	−0.01731 (19)	0.34639 (18)	0.0361 (5)	
C15	0.5234 (5)	−0.0524 (2)	0.44230 (19)	0.0473 (6)	
H15A	0.5004	−0.1314	0.4423	0.057*	
H15B	0.3827	−0.0158	0.4417	0.057*	
C16	0.6988 (5)	−0.0170 (3)	0.5398 (2)	0.0541 (7)	
H16A	0.7707	−0.0810	0.5769	0.065*	
H16B	0.6297	0.0249	0.5892	0.065*	
C17	0.8697 (4)	0.0546 (2)	0.49776 (19)	0.0403 (5)	
H17	0.9885	0.0051	0.4858	0.048*	
C18	0.5805 (4)	0.1853 (2)	0.3979 (2)	0.0424 (6)	
H18A	0.6635	0.2506	0.4224	0.064*	
H18B	0.4934	0.1638	0.4492	0.064*	
H18C	0.4837	0.1998	0.3304	0.064*	
C19	0.2822 (4)	0.1371 (2)	0.0358 (2)	0.0448 (6)	
H19A	0.3606	0.2053	0.0347	0.067*	
H19B	0.2153	0.1350	0.0977	0.067*	
H19C	0.1685	0.1313	−0.0276	0.067*	
C20	0.9805 (4)	0.1466 (2)	0.5690 (2)	0.0438 (6)	
C21	0.9195 (6)	0.1652 (3)	0.6749 (2)	0.0545 (7)	
H21A	1.0151	0.2205	0.7135	0.082*	
H21B	0.9364	0.0978	0.7150	0.082*	
H21C	0.7678	0.1894	0.6645	0.082*	

### Atomic displacement parameters (Å^2^)

**Table:**

	*U*^11^	*U*^22^	*U*^33^	*U*^12^	*U*^13^	*U*^23^
O1	0.0810 (15)	0.0551 (12)	0.0400 (9)	−0.0012 (12)	−0.0008 (9)	−0.0079 (9)
O2	0.0463 (10)	0.0431 (9)	0.0411 (9)	−0.0159 (8)	0.0053 (7)	0.0008 (8)
O3	0.0571 (11)	0.0313 (8)	0.0501 (10)	0.0073 (8)	0.0156 (8)	0.0067 (7)
O4	0.0806 (15)	0.0832 (17)	0.0480 (11)	−0.0383 (14)	0.0209 (10)	−0.0205 (10)
C1	0.0452 (13)	0.0503 (14)	0.0374 (12)	−0.0085 (12)	0.0109 (10)	0.0003 (11)
C2	0.0565 (16)	0.0568 (15)	0.0380 (12)	−0.0097 (14)	0.0106 (11)	0.0009 (11)
C3	0.0550 (15)	0.0472 (14)	0.0383 (12)	0.0041 (13)	0.0036 (11)	−0.0058 (11)
C4	0.0495 (14)	0.0386 (13)	0.0471 (13)	−0.0047 (11)	0.0047 (11)	−0.0069 (10)
C5	0.0405 (12)	0.0307 (11)	0.0441 (12)	−0.0020 (10)	0.0087 (10)	−0.0033 (9)
C6	0.0463 (14)	0.0444 (14)	0.0533 (14)	−0.0170 (12)	0.0108 (11)	−0.0031 (12)
C7	0.0540 (15)	0.0400 (13)	0.0491 (14)	−0.0182 (12)	0.0154 (11)	0.0018 (11)
C8	0.0374 (11)	0.0306 (10)	0.0412 (11)	−0.0044 (9)	0.0142 (9)	0.0002 (9)
C9	0.0316 (11)	0.0288 (10)	0.0362 (10)	−0.0017 (9)	0.0103 (8)	−0.0013 (8)
C10	0.0365 (12)	0.0303 (11)	0.0376 (11)	−0.0008 (9)	0.0092 (8)	−0.0005 (9)
C11	0.0372 (11)	0.0335 (11)	0.0393 (11)	−0.0070 (10)	0.0132 (9)	0.0013 (9)
C12	0.0329 (11)	0.0345 (12)	0.0391 (11)	−0.0029 (10)	0.0120 (9)	−0.0006 (9)
C13	0.0374 (11)	0.0324 (10)	0.0369 (11)	−0.0013 (10)	0.0129 (9)	−0.0017 (9)
C14	0.0384 (12)	0.0314 (11)	0.0416 (12)	−0.0031 (10)	0.0155 (9)	0.0008 (9)
C15	0.0569 (15)	0.0443 (13)	0.0441 (13)	−0.0121 (12)	0.0178 (11)	0.0053 (11)
C16	0.0656 (17)	0.0578 (16)	0.0401 (13)	−0.0111 (15)	0.0134 (12)	0.0057 (12)
C17	0.0461 (13)	0.0412 (12)	0.0343 (11)	−0.0013 (11)	0.0098 (9)	−0.0007 (10)
C18	0.0419 (13)	0.0385 (12)	0.0505 (13)	0.0019 (11)	0.0183 (10)	−0.0062 (10)
C19	0.0465 (13)	0.0331 (11)	0.0528 (14)	0.0037 (11)	0.0047 (11)	−0.0009 (11)
C20	0.0483 (13)	0.0439 (13)	0.0397 (12)	−0.0006 (12)	0.0098 (10)	−0.0033 (10)
C21	0.0740 (18)	0.0466 (15)	0.0462 (14)	0.0022 (14)	0.0200 (13)	−0.0081 (12)

### Geometric parameters (Å, °)

**Table:**

O1—C3	1.428 (3)	C9—C11	1.531 (3)
O1—H1	0.88 (5)	C9—C10	1.560 (3)
O2—C12	1.432 (3)	C9—H9	0.9800
O2—H2	0.80 (4)	C10—C19	1.530 (3)
O3—C14	1.440 (3)	C11—C12	1.519 (3)
O3—H3	0.81 (4)	C11—H11A	0.9700
O4—C20	1.200 (3)	C11—H11B	0.9700
C1—C2	1.528 (3)	C12—C13	1.533 (3)
C1—C10	1.538 (3)	C12—H12	0.9800
C1—H1A	0.9700	C13—C18	1.534 (3)
C1—H1B	0.9700	C13—C14	1.552 (3)
C2—C3	1.510 (4)	C13—C17	1.568 (3)
C2—H2A	0.9700	C14—C15	1.527 (3)
C2—H2B	0.9700	C15—C16	1.536 (4)
C3—C4	1.510 (4)	C15—H15A	0.9700
C3—H3A	0.9800	C15—H15B	0.9700
C4—C5	1.532 (3)	C16—C17	1.539 (4)
C4—H4A	0.9700	C16—H16A	0.9700
C4—H4B	0.9700	C16—H16B	0.9700
C5—C6	1.515 (3)	C17—C20	1.513 (4)
C5—C10	1.546 (3)	C17—H17	0.9800
C5—H5	0.9800	C18—H18A	0.9600
C6—C7	1.521 (4)	C18—H18B	0.9600
C6—H6A	0.9700	C18—H18C	0.9600
C6—H6B	0.9700	C19—H19A	0.9600
C7—C8	1.528 (3)	C19—H19B	0.9600
C7—H7A	0.9700	C19—H19C	0.9600
C7—H7B	0.9700	C20—C21	1.489 (4)
C8—C14	1.532 (3)	C21—H21A	0.9600
C8—C9	1.536 (3)	C21—H21B	0.9600
C8—H8	0.9800	C21—H21C	0.9600
			
C3—O1—H1	103 (3)	C12—C11—C9	114.89 (18)
C12—O2—H2	106 (3)	C12—C11—H11A	108.5
C14—O3—H3	102 (3)	C9—C11—H11A	108.5
C2—C1—C10	113.85 (19)	C12—C11—H11B	108.5
C2—C1—H1A	108.8	C9—C11—H11B	108.5
C10—C1—H1A	108.8	H11A—C11—H11B	107.5
C2—C1—H1B	108.8	O2—C12—C11	106.34 (17)
C10—C1—H1B	108.8	O2—C12—C13	113.46 (19)
H1A—C1—H1B	107.7	C11—C12—C13	111.22 (18)
C3—C2—C1	111.3 (2)	O2—C12—H12	108.6
C3—C2—H2A	109.4	C11—C12—H12	108.6
C1—C2—H2A	109.4	C13—C12—H12	108.6
C3—C2—H2B	109.4	C12—C13—C18	110.96 (19)
C1—C2—H2B	109.4	C12—C13—C14	106.61 (18)
H2A—C2—H2B	108.0	C18—C13—C14	112.93 (19)
O1—C3—C4	112.1 (2)	C12—C13—C17	117.45 (19)
O1—C3—C2	108.8 (2)	C18—C13—C17	109.21 (19)
C4—C3—C2	110.5 (2)	C14—C13—C17	99.21 (18)
O1—C3—H3A	108.5	O3—C14—C15	108.5 (2)
C4—C3—H3A	108.5	O3—C14—C8	107.79 (18)
C2—C3—H3A	108.5	C15—C14—C8	117.80 (19)
C3—C4—C5	110.8 (2)	O3—C14—C13	107.04 (18)
C3—C4—H4A	109.5	C15—C14—C13	103.07 (18)
C5—C4—H4A	109.5	C8—C14—C13	112.14 (19)
C3—C4—H4B	109.5	C14—C15—C16	104.3 (2)
C5—C4—H4B	109.5	C14—C15—H15A	110.9
H4A—C4—H4B	108.1	C16—C15—H15A	110.9
C6—C5—C4	112.7 (2)	C14—C15—H15B	110.9
C6—C5—C10	112.1 (2)	C16—C15—H15B	110.9
C4—C5—C10	112.28 (19)	H15A—C15—H15B	108.9
C6—C5—H5	106.4	C15—C16—C17	107.0 (2)
C4—C5—H5	106.4	C15—C16—H16A	110.3
C10—C5—H5	106.4	C17—C16—H16A	110.3
C5—C6—C7	111.4 (2)	C15—C16—H16B	110.3
C5—C6—H6A	109.4	C17—C16—H16B	110.3
C7—C6—H6A	109.4	H16A—C16—H16B	108.6
C5—C6—H6B	109.4	C20—C17—C16	117.6 (2)
C7—C6—H6B	109.4	C20—C17—C13	114.9 (2)
H6A—C6—H6B	108.0	C16—C17—C13	103.5 (2)
C6—C7—C8	111.8 (2)	C20—C17—H17	106.7
C6—C7—H7A	109.3	C16—C17—H17	106.7
C8—C7—H7A	109.3	C13—C17—H17	106.7
C6—C7—H7B	109.3	C13—C18—H18A	109.5
C8—C7—H7B	109.3	C13—C18—H18B	109.5
H7A—C7—H7B	107.9	H18A—C18—H18B	109.5
C7—C8—C14	112.00 (19)	C13—C18—H18C	109.5
C7—C8—C9	112.19 (19)	H18A—C18—H18C	109.5
C14—C8—C9	108.93 (17)	H18B—C18—H18C	109.5
C7—C8—H8	107.8	C10—C19—H19A	109.5
C14—C8—H8	107.8	C10—C19—H19B	109.5
C9—C8—H8	107.8	H19A—C19—H19B	109.5
C11—C9—C8	110.64 (17)	C10—C19—H19C	109.5
C11—C9—C10	113.70 (18)	H19A—C19—H19C	109.5
C8—C9—C10	112.37 (17)	H19B—C19—H19C	109.5
C11—C9—H9	106.5	O4—C20—C21	119.9 (3)
C8—C9—H9	106.5	O4—C20—C17	120.5 (2)
C10—C9—H9	106.5	C21—C20—C17	119.6 (2)
C19—C10—C1	109.6 (2)	C20—C21—H21A	109.5
C19—C10—C5	112.09 (18)	C20—C21—H21B	109.5
C1—C10—C5	107.21 (19)	H21A—C21—H21B	109.5
C19—C10—C9	111.28 (19)	C20—C21—H21C	109.5
C1—C10—C9	109.66 (17)	H21A—C21—H21C	109.5
C5—C10—C9	106.91 (18)	H21B—C21—H21C	109.5
			
C10—C1—C2—C3	−55.5 (3)	C11—C12—C13—C18	−66.8 (2)
C1—C2—C3—O1	178.7 (2)	O2—C12—C13—C14	176.39 (17)
C1—C2—C3—C4	55.3 (3)	C11—C12—C13—C14	56.5 (2)
O1—C3—C4—C5	−178.8 (2)	O2—C12—C13—C17	−73.5 (3)
C2—C3—C4—C5	−57.3 (3)	C11—C12—C13—C17	166.6 (2)
C3—C4—C5—C6	−173.2 (2)	C7—C8—C14—O3	68.6 (2)
C3—C4—C5—C10	59.0 (3)	C9—C8—C14—O3	−56.1 (2)
C4—C5—C6—C7	173.0 (2)	C7—C8—C14—C15	−54.5 (3)
C10—C5—C6—C7	−59.2 (3)	C9—C8—C14—C15	−179.1 (2)
C5—C6—C7—C8	53.6 (3)	C7—C8—C14—C13	−173.83 (19)
C6—C7—C8—C14	−174.2 (2)	C9—C8—C14—C13	61.5 (2)
C6—C7—C8—C9	−51.3 (3)	C12—C13—C14—O3	55.5 (2)
C7—C8—C9—C11	−177.7 (2)	C18—C13—C14—O3	177.62 (18)
C14—C8—C9—C11	−53.1 (2)	C17—C13—C14—O3	−66.9 (2)
C7—C8—C9—C10	54.0 (2)	C12—C13—C14—C15	169.8 (2)
C14—C8—C9—C10	178.61 (19)	C18—C13—C14—C15	−68.1 (2)
C2—C1—C10—C19	−67.9 (3)	C17—C13—C14—C15	47.4 (2)
C2—C1—C10—C5	53.9 (3)	C12—C13—C14—C8	−62.5 (2)
C2—C1—C10—C9	169.6 (2)	C18—C13—C14—C8	59.6 (2)
C6—C5—C10—C19	−63.2 (3)	C17—C13—C14—C8	175.10 (18)
C4—C5—C10—C19	64.9 (3)	O3—C14—C15—C16	77.3 (3)
C6—C5—C10—C1	176.6 (2)	C8—C14—C15—C16	−160.0 (2)
C4—C5—C10—C1	−55.4 (2)	C13—C14—C15—C16	−35.9 (3)
C6—C5—C10—C9	59.0 (2)	C14—C15—C16—C17	9.7 (3)
C4—C5—C10—C9	−172.89 (18)	C15—C16—C17—C20	147.8 (2)
C11—C9—C10—C19	−60.2 (2)	C15—C16—C17—C13	19.9 (3)
C8—C9—C10—C19	66.4 (2)	C12—C13—C17—C20	75.4 (3)
C11—C9—C10—C1	61.2 (2)	C18—C13—C17—C20	−52.0 (3)
C8—C9—C10—C1	−172.2 (2)	C14—C13—C17—C20	−170.3 (2)
C11—C9—C10—C5	177.11 (18)	C12—C13—C17—C16	−155.0 (2)
C8—C9—C10—C5	−56.2 (2)	C18—C13—C17—C16	77.6 (3)
C8—C9—C11—C12	51.3 (2)	C14—C13—C17—C16	−40.8 (2)
C10—C9—C11—C12	178.83 (19)	C16—C17—C20—O4	177.7 (3)
C9—C11—C12—O2	−177.92 (18)	C13—C17—C20—O4	−60.1 (3)
C9—C11—C12—C13	−53.9 (2)	C16—C17—C20—C21	−0.7 (4)
O2—C12—C13—C18	53.0 (3)	C13—C17—C20—C21	121.5 (3)

### Hydrogen-bond geometry (Å, °)

**Table:**

*D*—H···*A*	*D*—H	H···*A*	*D*···*A*	*D*—H···*A*
O3—H3···O4^i^	0.81 (4)	2.19 (4)	2.916 (3)	150 (3)
O2—H2···O4	0.80 (4)	2.01 (4)	2.771 (3)	158 (3)
O1—H1···O2^ii^	0.88 (5)	2.05 (6)	2.928 (3)	170 (4)

Symmetry codes: (i) −*x*+2, *y*−1/2, −*z*+1; (ii) −*x*+1, *y*−1/2, −*z*.
